# Relief of Urinary Urgency, Hesitancy, and Male Pelvic Pain with Pulse Radiofrequency Ablation of the Pudendal Nerve: A Case Presentation

**DOI:** 10.1155/2013/125703

**Published:** 2013-03-28

**Authors:** Christopher Bui, Sanjog Pangarkar, Scott I. Zeitlin

**Affiliations:** ^1^Department of Physical Medicine and Rehabilitation, West Los Angeles Veterans Administration/UCLA, Los Angeles, CA 90073, USA; ^2^Department of Physical Medicine and Rehabilitation, West Los Angeles Veterans Administration, California Pain Medicine Centers, Los Angeles, CA 90073, USA; ^3^Department of Urology, Department of Ob/Gyn, David Geffen School of Medicine at UCLA, Los Angeles, CA 90073, USA

## Abstract

*Background and Aims*. This report demonstrates the utility of a pudendal nerve block by pulsed radiofrequency ablation (RFA) for the treatment of male pelvic pain and urinary urgency and hesitancy. *Methods*. The patient is an 86-year-old gentleman with a 30-year history of urinary hesitancy and urgency. The patient also had pain in the area of the perineum but considered it a secondary issue. The patient was seen by a number of specialists, tried various medications, and underwent a variety of procedures to no avail. Therefore, the patient underwent a pulsed RFA of the pudendal nerve. *Results*. The patient underwent a pulsed RFA of the pudendal nerve; the patient reported marked improvement in his pelvic pain as well as a drastic reduction in his urinary urgency and hesitancy. *Conclusion*. Urinary urgency and hesitancy and male pelvic pain are some of the most common symptoms affecting men. Pudendal nerve block by pulsed RFA is an effective treatment of pelvic pain. It may also hold some therapeutic value in the treatment of urinary urgency and hesitancy as our case demonstrated. Further studies are needed to help clarify both the anatomy of the pelvis as well as if pudendal blocks are effective in treating more than pelvic pain.

## 1. Introduction

This paper demonstrates the utility of a pudendal nerve block by pulsed radiofrequency ablation (RFA) for the treatment of male pelvic pain, urinary urgency and hesitancy. We describe the case of a man who presented with the above symptoms. The patient was seen by a number of specialists, tried various medications, and underwent a variety of procedures to no avail. However, after pulsed RFA of the pudendal nerve, the patient reported marked improvement in his pelvic pain as well as a drastic reduction in his urinary urgency and hesitancy.

## 2. Anatomy of the Pudendal Nerve

**Figure 1:**
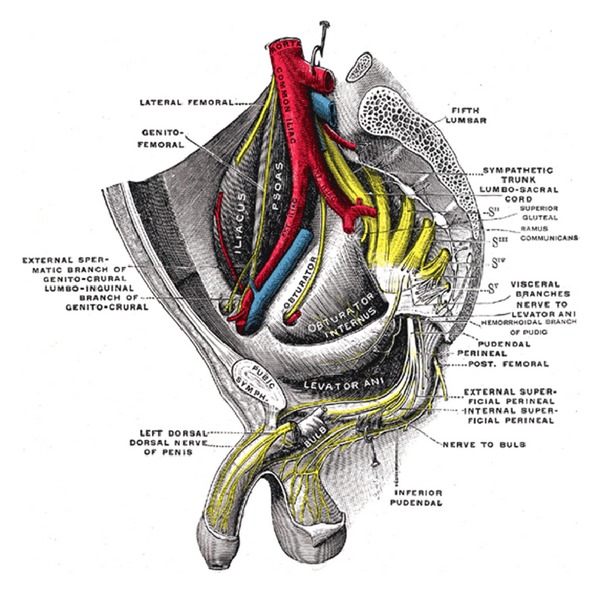
Pudendal nerve anatomy. Gray's anatomy, 2011.

The pudendal nerve originates in the sacral plexus from fibers of the second, third, and fourth anterior sacral rami (S2,3,4). The nerve follows a complex course and travels behind the sacrospinous ligament, medial to the ischial spine. The nerve exits the pelvis through the greater sciatic foramen and enters the ischiorectal fossa via the lesser sciatic foramen. After traveling through the pudendal canal (Alcock's canal), it divides into its terminating branches [1, 2] (Figure 1).

## 3. Case Presentation

The patient is a 86-year-old gentleman with a 30-year history of urinary hesitancy and urgency. The patient also had pain in the area of the perineum but considered it a secondary issue.

In 1953, while in the Navy, the patient was told that he had an enlarged prostate. It was not until 20 years later that the patient began to have significant symptoms of urinary hesitancy, urgency, and frequency. He noted that he woke 3-4 times per night to urinate. His symptoms appeared to be cyclical in nature where they would improve and then again worsen. He tried a number of medications including terazosin, dutasteride, pentosan, bethanechol, and onabotulinum toxin A with minimal effect.

Before visiting our clinic, the patient underwent a cystoscopy as well as an urodynamic evaluation that eventually led to the first of three transurethral resections of the prostate (TURP). By 2011, the patient also underwent a number of procedures, which included a transrectal ultrasound with a block of the pudendal nerve and an onabotulinum toxin A injection directly into the prostate. Both procedures only produced short-term symptomatic relief.

On physical examination, the patient had a normal appearance and was in no acute distress. Neurologically, patient's cranial nerve exam was grossly intact. There was a positive Hoffmann's reflex on the right. Otherwise, the patient's exam was normal.

**Figure 2:**
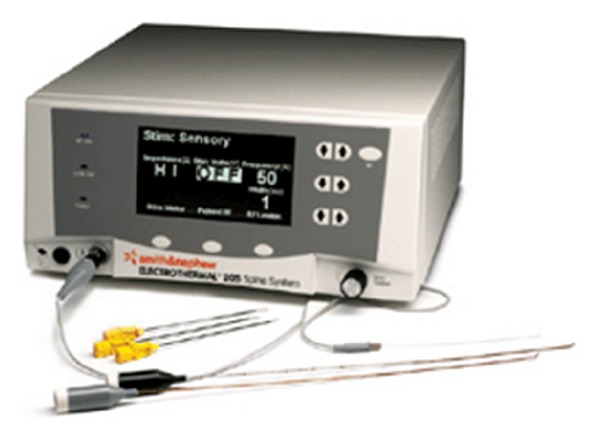
Smith & Nephew Electrothermal 20S.

**Figure 3:**
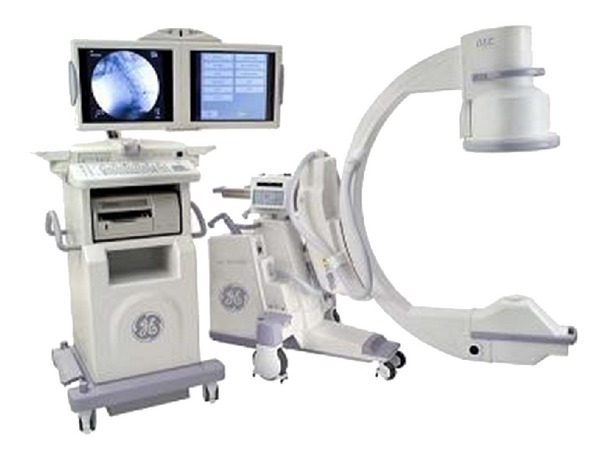
GE OCE 9900 Elite C-arm Fluoroscope.

He was initially diagnosed with pelvic floor dyssynergia as well as concurrent neuropathy of the pelvic nerves. We planned to start the patient on an anticonvulsant as well as a referral to a physical therapist for pelvic floor strengthening. He was started on pregabalin and agreed to undergo a pudendal block with pulsed radiofrequency ablation (RFA). Fluoroscopic guidance was used to both view the patient's anatomy as well as direct the RF needle (Figure 3). The needle was inserted over the ischium and advanced until the ischial bone was reached. At that point, a pulsed radiofrequency burn was performed for two minutes at 40 degrees C, utilizing the Smith & Nephew Electrothermal 20S Spine System (Figure 2). This was performed at two locations on each side for a total of 4 minutes of pulsed RF. To conclude the procedure, the nerve was blocked with 20 mg of methylprednisolone and a mixture of 1 mL of 0.25% Marcaine and 1 mL of 1% lidocaine.

The patient was seen for a followup one week after the pudendal nerve block. He noted that his pelvic pain was much improved and the urinary hesitancy and urgency were much reduced. In addition, his sleep duration and quality was improved due to a reduction in his nocturia to 0-1 time nightly. 

## 4. Discussion

Urinary urgency and hesitancy are some of the most common irritative voiding symptoms [3]. These symptoms are often the result of bladder outlet obstruction leading to the aberrant flow of urine out from the bladder. However, when there appears to be no such pathology, the treatment becomes difficult and ill-directed. Paired with male pelvic pain these symptoms are extremely bothersome to the daily function of the patient.

Our patient's symptoms were not only affecting his urologic function but more importantly his quality of life. The only treatment that provided significant relief from his symptoms of pelvic pain, urinary urgency and hesitancy was a pudendal nerve block by pulsed RFA.

The pudendal nerve supplies both sensory and somatic components to the penis and clitoris, bulbospongiosus and ischiocavernosus muscles, and areas around the scrotum, perineum, and anus. Blocking the nerve severs pain signals from the pelvic region [3–5]. This effect was appreciated in our patient in addition to an improvement in his urinary urgency and hesitancy.

With regards to the control of micturition, the detrusor muscle and internal urethral sphincter are made of smooth muscles and are under autonomic control. Prohibiting the flow of urine and bladder emptying are functions primarily controlled by these muscles. Both muscles derive their sympathetic innervation from the hypogastric nerve (T10-L2) and their parasympathetic innervations from the pelvic plexus (S2-S4). The external urethral sphincter is a skeletal muscle that is located distal to the prostate. It is the secondary sphincter that controls the flow of urine and is innervated by the dorsal nerve of the penis, a terminal branch of the pudendal nerve. Therefore, blocking the pudendal nerve may inhibit the action of the external urethral sphincter and cause inability to control urine flow. Our patient reported vast improvement in his urinary urgency and hesitancy. This leads us to believe that the pudendal nerve may also provide some innervation to the detrusor muscle and/or internal urethral sphincter (Table 1). Further anatomic studies and nerve mapping would be extremely helpful in answering this question [1, 2, 6–9].

## 5. Conclusion

Urinary urgency and hesitancy and male pelvic pain are some of the most common symptoms affecting men. Pudendal nerve block by pulsed RFA is an effective treatment of pelvic pain. It may also hold some therapeutic value in the treatment of urinary urgency and hesitancy as our case demonstrated. Further studies are needed to help clarify both the anatomy of the pelvis as well as if pudendal blocks are effective in treating more than pelvic pain.

## Figures and Tables

**Table 1:** Terminal branches and anatomic locations [1, 2, 6, 7].

Branch	Anatomic description
Inferior anal nerves	Develop after passing through the greater sciatic foramen
Perineal nerve	Superficial terminal branch
Dorsal nerve of the penis/clitoris	Deep terminal branch, diving into the deep perineal pouch. Innervates the external urethral sphincter
Posterior scrotal nerves/labial nerves	Innervates the posterior scrotum/labia

## Keys

RFA: radiofrequency ablation
TURP: transurethral resections of the prostate
T: thoracic
S: sacral.
